# Acute retinal necrosis in a patient on immunosuppressive treatment for COVID-19 pneumonia: a case report

**DOI:** 10.1186/s12886-022-02692-5

**Published:** 2022-11-30

**Authors:** Takeyuki Nishiyama, Hiroki Tsujinaka, Yutaro Mizusawa, Tetsuo Ueda, Nahoko Ogata

**Affiliations:** Department of Ophthalmology, Nara Medical University, 840 Shijo-cho, Kashihara, Nara, 634-8522 Japan

**Keywords:** Epstein–Barr virus, Uveitis, Acute retinal necrosis, COVID-19, Pneumonia, Immunosuppression

## Abstract

**Background:**

Patients with coronavirus disease 2019 (COVID-19) occasionally develop ocular complications. We report a case of acute retinal necrosis (ARN) caused by Epstein–Barr Virus (EBV) that developed in a patient who had severe acute respiratory syndrome due to SARS-CoV-2 infection.

**Case presentation:**

A 68-year-old woman complained of floaters and blurred vision in her right eye as she was receiving systemic prednisolone for COVID-19 pneumonia under isolation in our hospital. The patient visited an ophthalmologist following her discharge from the hospital and after the 2 weeks of isolation had ended. At the initial examination, her best-corrected visual acuity (BCVA) was 20/100 in the right eye, and the eye showed moderate anterior segment inflammation and vitreous opacities. Treatment was initiated with topical 0.1% betamethasone and 1.5% levofloxacin. After 1 month, the inflammation in the right eye decreased and her BCVA improved to 20/40. However, on day 48 from her initial visit, the inflammation in her right eye worsened and her BCVA decreased to 20/2000 by day 80. Pars plana vitrectomy with silicone oil tamponade was performed to remove the vitreous opacities, and expanded white exudates peripherally and retinal vessels with white sheathing suggestive of acute retinal necrosis (ARN) were seen intraoperatively. Analysis of the vitreous sample revealed EBV positivity on polymerase chain reaction. The patient was diagnosed with EBV-associated ARN and treated with systemic steroids and valaciclovir. The ocular inflammation gradually decreased, and she was discharged from the hospital. However, a week later, the inflammation in the right eye markedly worsened. Despite another course of steroids, the inflammation worsened, resulting in total retinal detachment and absolute glaucoma. Because of the severe pain, the right eye was enucleated.

**Conclusions:**

Clinicians should be aware that COVID-19 and immunosuppressive treatment can reactivate EBV in the eye.

**Supplementary Information:**

The online version contains supplementary material available at 10.1186/s12886-022-02692-5.

## Background

The pandemic due to severe acute respiratory syndrome coronavirus 2 (SARS-CoV-2) has had health implications of unprecedented magnitude. The disease can affect every organ of the body.

Patients with coronavirus disease 2019 (COVID-19) occasionally develop ocular complications, and various manifestations in the eye directly or indirectly associated with the virus have been reported, i.e, conjunctivitis, retinal hemorrhage, disc edema, uveitis, vitritis, and retinitis [1, 2].

Ophthalmologists should be aware of the possible association of ocular disease in patients affected by COVID-19 because ophthalmic manifestations may be the presenting feature of COVID-19 or they may develop several weeks after recovery.

Isolation of COVID-19-affected patients may delay opportunities for eye examination and worsen eye inflammation, resulting in a delay in treatment and difficulties in diagnosis.

We report a case of acute retinal necrosis (ARN) in a patient on immunosuppressive treatment for COVID-19 pneumonia in whom Epstein–Barr virus (EBV) was detected from the vitreous.

## Case presentation

A 68-year-old woman presented with floaters in her right eye. Her review of systems showed that she had cough, fever, and shortness of breath that had started 2 weeks earlier, and her symptoms had worsened after a week. She visited the hospital and was diagnosed with COVID-19 pneumonia by general examination and polymerase chain reaction (PCR). She had not been vaccinated against COVID-19. She had undergone surgery for breast cancer 2 years prior and the cancer treatment had ended. She was not on any medication before she acquired COVID-19. She received systemic antibiotics and remdesivir for COVID-19 pneumonia and was in isolation at our hospital.

Systemic prednisolone treatment was also started from 0.6 mg/kg/day and tapered gradually for 14 days. The patient was under systemic treatment in isolation when she presented with floaters in her right eye.

Five days after finishing prednisolone treatment, she was discharged from the hospital. She complained of worsening floaters and blurred vision but she was required to self-isolate for 2 weeks, thus she visited an ophthalmologist 3 weeks after the primary ocular symptoms.

**Fig. 1:**
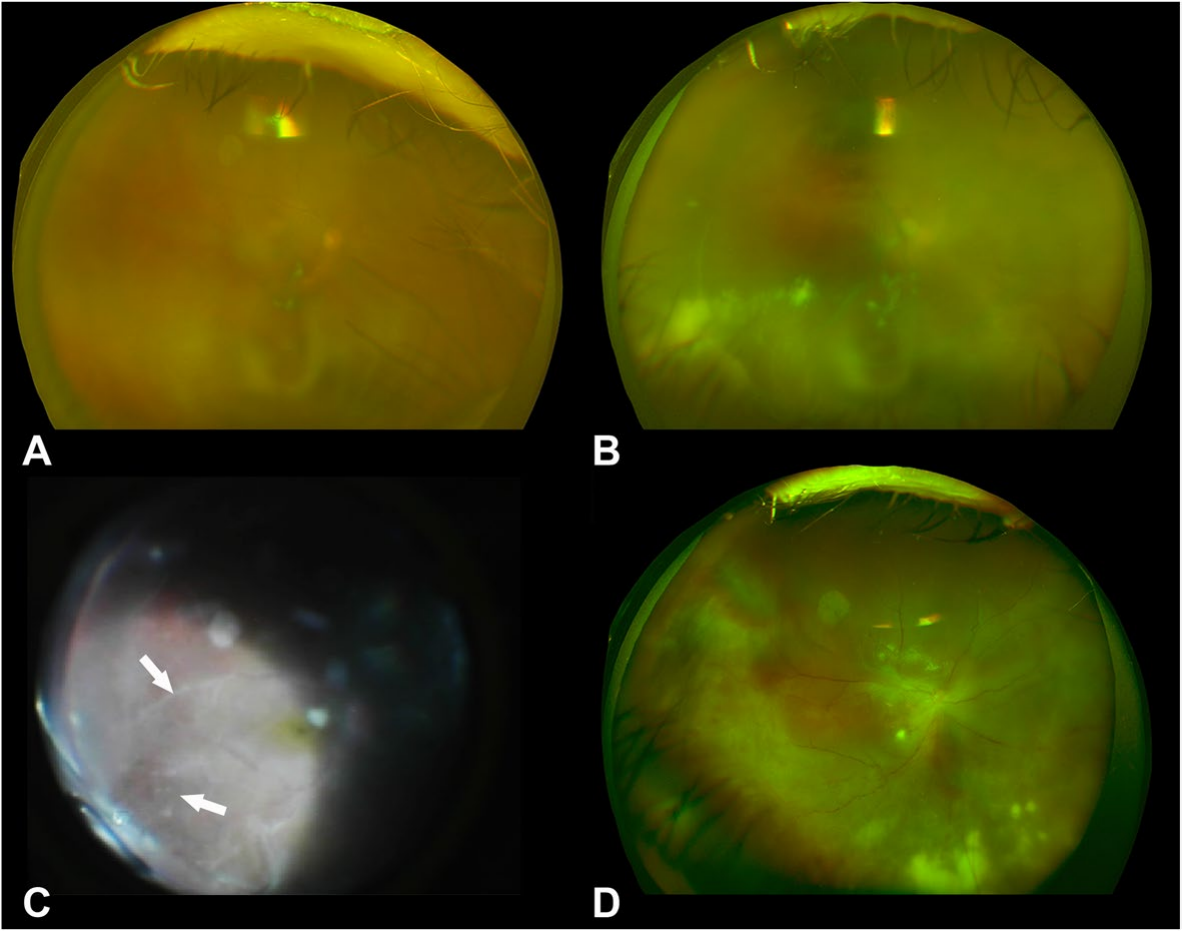
**A** Fundus photograph at the initial examination. The fundus is veiled because of moderate vitreous opacities. **B** Fundus photograph at day 80 showing vitreous opacities and yellowish-white retinal exudates. **C** Intraoperative fundus photograph during pars plana vitrectomy showing peripheral retinitis with white exudates and white retinal vessels with sheathing. **D** Fundus photograph on postoperative day 30 showing residual retinal exudates but no obvious signs of a progression of the retinitis

**Fig. 2:**
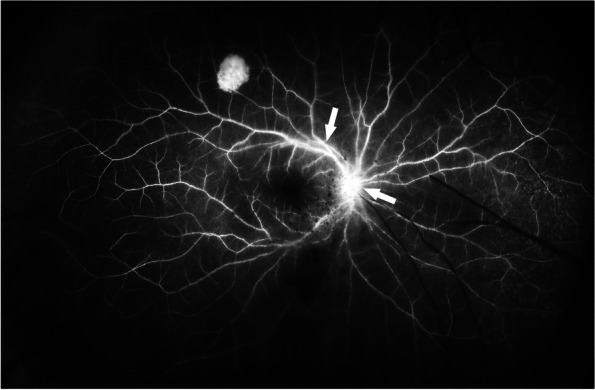
Fluorescein angiogram at the initial visit showing leakage at the optic nerve head and vessels of the vascular arcades

At the initial visit, she reported an increase in the floaters and blurred vision in the right eye. Her best-corrected visual acuity (BCVA) was 20/100 in the right eye and 20/16 in the left eye. The intraocular pressure was 16 mmHg in the right eye and 17 mmHg in the left eye. No significant findings were noted in the left eye. Slit-lamp examination of the right eye revealed grade 2+ cells in the anterior chamber and posterior synechia. Fundus examination showed vitreous opacities and retinal exudates (Fig. 1A). Fluorescein angiography showed leakage from the optic disc and the vessels of the vascular arcades (Fig. 2).

Serological test results indicated only mild systemic inflammatory changes, and the β-D-glucan level that suggests a fungal infection was low (Table 1). Multiplex PCR of aqueous humor samples was performed commercially (Catalog No. 8665-6, SRL, Inc., Tokyo, Japan). The examined viruses and the used primers by PCR are shown in Supplemental Table 1. Bacterial and fungal cultures of aqueous humor were also performed. No pathogens were identified. Both the ophthalmologists and the general physicians, including rheumatologists, assessed the patient to identify any systemic causes. No systemic cause of the uveitis was identified.

**Table 1:** Results of serological tests

Laboratory parameters	Patient Values	Reference range
White blood cells	5000 μL	3300 – 8600 μL
CRP	0.44 mg/dL	< 0.14 mg/dL
KL-6	1060 U/mL	< 500 U/mL
soluble IL-2 receptor	552 U/mL	145 – 519 U/mL
β-D-glucan	7.3 pg/mL	< 11.0 pg/mL
ACE	12.8 U/L	7.0 – 25.0 U/L
Treponema pallidum hemagglutination test	Negative	< 16.0 U/mL
HSV antibody complement fixation test	16 times	< 4 times
VZV antibody complement fixation test	4 times	< 4 times
Toxoplasma IgG antibody	115 U/mL	< 6 U/mL
Toxoplasma IgM antibody	0.4 C.O. I	< 0.8 C.O. I
Antinuclear antibody	Negative	< 40 times

*CRP* C-reactive protein, *KL-6* Krebs von den Lungen-6, *ACE* angiotensin-converting enzyme, *HSV* herpes simplex virus, *VZV* varicella-zoster virus, *IgG* immunoglobulin G, *IgM* immunoglobulin M

**Fig. 3:**
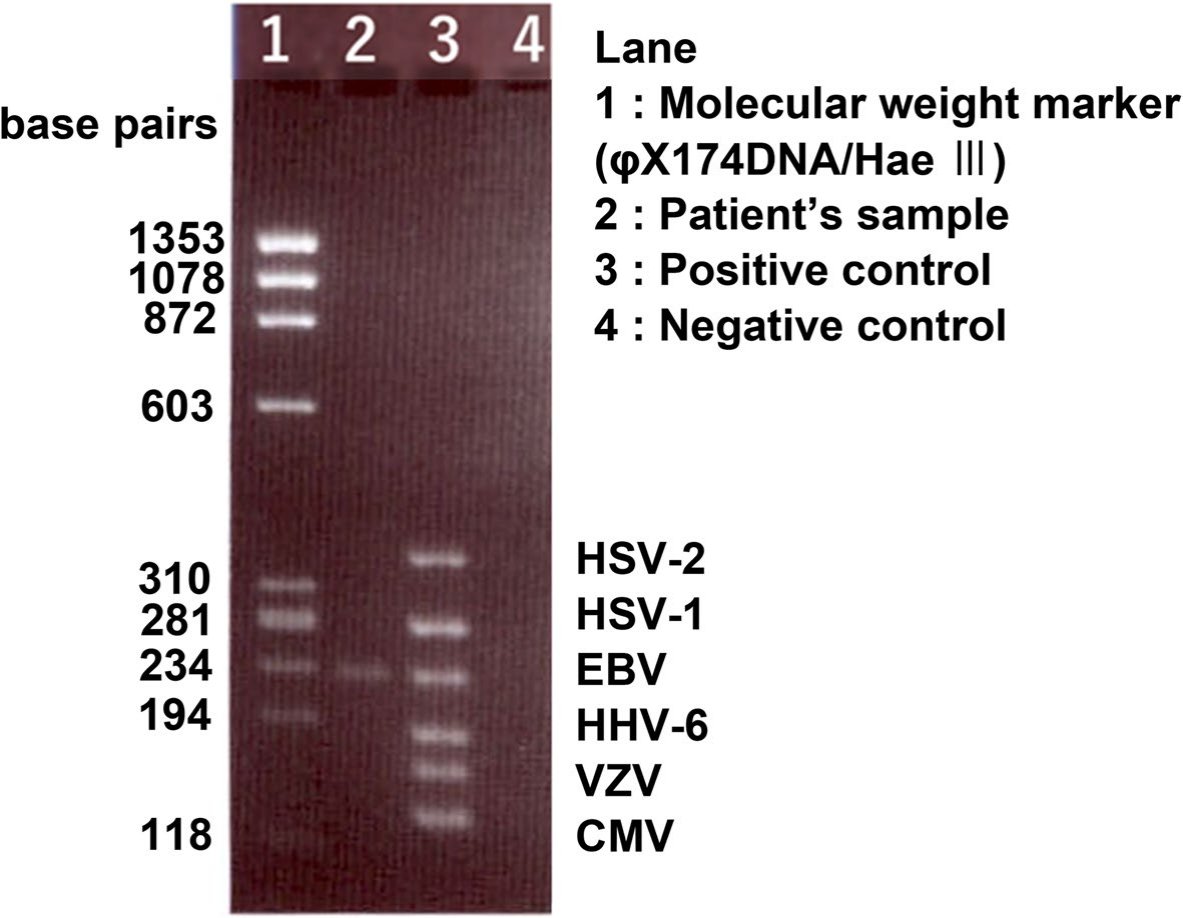
Results of multiplex polymerase chain reaction of the vitreous sample showing the presence of Epstein–Barr virus DNA. For the electrophoresis, we used 3% agarose gel and sterile water was loaded in the negative control lane (Lane 4). Positive controls were for HSV-2, HSV-1, EBV, HHV-6, VZV, and CMV. HSV-2: herpes simplex virus type2, HSV-1: herpes simplex virus type1, EBV: Epstein–Barr virus, HHV-6: Human herpesvirus 6, VZV: varicella-zoster virus, and CMV: cytomegalovirus

The patient was treated with topical 0.1% betamethasone 4 times/day in the right eye for 30 days. Topical 1.5% levofloxacin 4 times/day was also prescribed to prevent secondary bacterial infection. After the treatment, slit-lamp examination of the right eye revealed grade 1+ cells in the anterior chamber and the vitreous opacities and retinal exudates were also attenuated after the treatment. The BCVA improved to 10/20. However, on day 48 after the initial eye examination, the inflammation worsened (Fig. 1B). The BCVA of the right eye gradually worsened during the time and finally decreased to 20/2000 by day 80. The fundus was not visible due to vitreous haze. Pars plana vitrectomy with silicone oil tamponade was performed to remove the vitreous opacities, and aqueous and vitreous samples were collected. Intraoperatively, dense opacities were found throughout the vitreous cavity. Expanded white exudates peripherally and retinal vessels with white sheathing suggestive of ARN were seen intraoperatively (Fig. 1C). The vitreous sample was examined by multiplex PCR (Catalog No. 8665-6, SRL, Inc., Tokyo, Japan) again, smear preparation, bacterial susceptibility tests, and bacterial and fungal cultures. No bacteria or fungi, including *Candida albicans*, were detected. On postoperative day 7, EBV was detected in the vitreous by multiplex PCR (Fig. 3), thus, the patient was diagnosed with EBV-related ARN.

She was treated with 1.25 mg/kg/day of systemic prednisolone and 3000 mg/day of systemic valaciclovir for the ARN. The inflammation in the eye gradually decreased (Fig. 1D), and on postoperative day 30, the prednisolone was tapered to 30 mg/day. The patient was then discharged from the hospital.

**Fig. 4:**
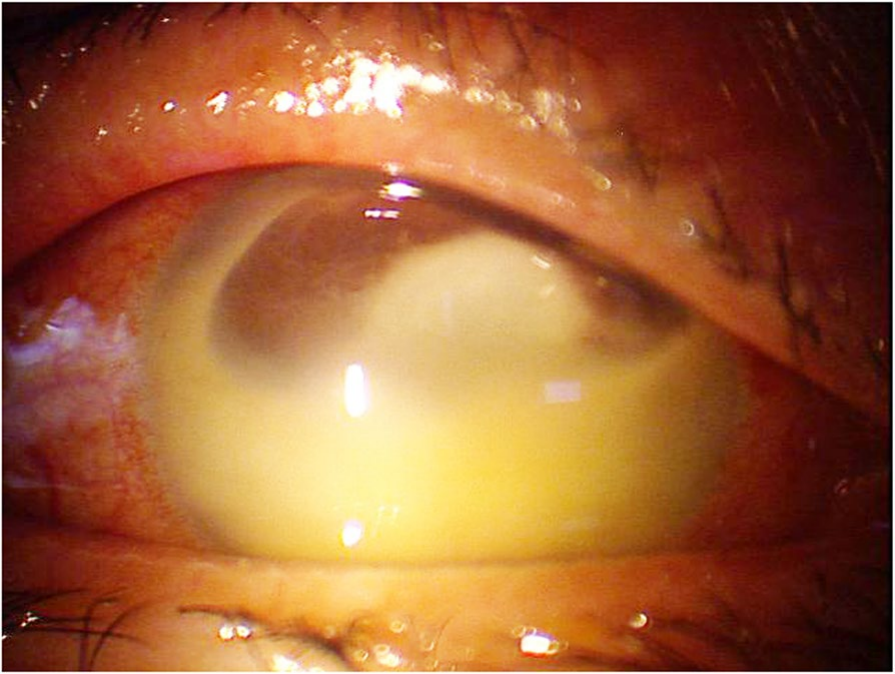
Photograph of the anterior segment on postoperative day 49 with hypopyon and posterior synechia

However, there was marked worsening of the inflammation with the development of hypopyon on postoperative day 35. Although prednisolone (1.25 mg/kg/day) was re-administered, the inflammation progressed, leading to the development of total retinal detachment and absolute glaucoma (Fig. 4). The right eye was enucleated on postoperative day 50 because of the severe pain and to prevent the spread of the infection to the extraocular tissues. EBV was not detected by EBV-encoded small RNA in situ hybridization in the retina of the enucleated eye, but histopathological examination of revealed *Candida albicans* in the vitreous and retina. Because there were no signs of *Candida* infection, except in the right eye, systemic treatment for *Candida* was not administered. The dose of prednisolone was gradually reduced, and all treatment was terminated.

## Discussion and Conclusions

EBV, also known as human herpesvirus 4, is a member of the herpesvirus family, and most individuals are infected by EBV during childhood. It is a well-known cause of infectious mononucleosis [3]. EBV is also recognized as an oncovirus, involved in the pathogenesis of many types of cancers, including malignant lymphoma, gastric cancer, and laryngeal cancer [4]. In contrast, EBV can be detected in eyes with severe inflammation, but ocular EBV infections, manifesting as uveitis, vitritis, and optic disk vasculitis, are rare [5–7]. In our case, comprehensive tests, including PCR, bacterial and fungal cultures of aqueous humor and vitreous samples, and serological tests, revealed no endogenous infective cause of the uveitis. Multiplex PCR tests of vitreous samples obtained during vitrectomy detected only the DNA of EBV, but bacterial and fungal detections were negative. The patient presented with unilateral uveitis, and the clinical findings during vitrectomy were consistent with ARN, i.e., dense opacities, expanded white exudates peripherally, retinal vascular arteriolitis, and no findings suggestive of fungal infection [8]. Moreover, the serum β-D-glucan level was low, thus *Candida* infection was ruled out. Based on these findings, the patient was eventually diagnosed with EBV-related ARN.

Few cases of ARN caused by EBV have been reported where EBV was detected in vitreous samples by PCR [9–11]. It has been suggested that EBV uveitis develops more frequently in patients with autoimmune diseases such as rheumatoid arthritis and systemic lupus erythematosus [9–12]. However, it has not been determined whether EBV reactivation is triggered by systemic hyperimmunity due to autoimmune diseases, or by immunosuppression due to systemic treatment for autoimmune diseases.

COVID-19 can induce a state of hyperimmunity [13–15]. In our case, it is not known whether the trigger for the ocular EBV infection was hyperimmunity due to the COVID-19 or the systemic steroid treatment for pneumonia that was immunosuppressive. A patient who is on corticosteroids will have a higher risk of becoming immunocompromised, with secondary reactivation of EBV. In this case, corticosteroid treatment for COVID-19 pneumonia could have induced an immunocompromised status. In addition, ocular examination and treatment might be delayed because of isolation due to COVID-19. These factors are assumed to have contributed to disease progression.

There is no established treatment for EBV uveitis. Therefore, our patient was treated with steroids and valaciclovir as reported [14]. This resulted in temporary resolution of the inflammation. However, systemic steroids may lead to further immunosuppression, resulting in additional infections. In this case, histopathological examination of the enucleated eye revealed *Candida albicans*.

In conclusion, we have presented a rare case of EBV-related ARN. The intensive treatment resulted in a temporary resolution of the inflammation. However, systemic steroids may lead to further immunosuppression, resulting in additional infections. Clinicians should be aware that COVID-19 and treatment can reactivate EBV in the eye.

## Supplementary Information

**Additional file 1.**

## Data Availability

Not applicable.

## Abbreviations

COVID-19: coronavirus disease 2019
EBV: Epstein–Barr virus
BCVA: best-corrected visual acuity
PCR: polymerase chain reaction
ARN: acute retinal necrosis
