# Supplementary material for: Acute retinal necrosis in a patient on immunosuppressive treatment for COVID-19 pneumonia: a case report
Source: BMC Ophthalmol. 2022 Nov 30;22:462. doi: 10.1186/s12886-022-02692-5 (PMC9709381; doi:10.1186/s12886-022-02692-5)
Supplement: Supplementary file 1 — Additional file 1. [file 12886_2022_2692_MOESM1_ESM.docx]

Supplemental Table 1

| Virus | Primer Sequence (F:sence R:anti-sence) | Positive control |
| --- | --- | --- |
| HSV-1 | F: TGT TGG CCT TCA TGA CCC TTG TGA AA | HSV-1 infected VR-3 cells |
|  | R: TAG CTC GAG AGC TTG ATC TTG TCG GTT |  |
| HSV-2 | F: AGT CCC ACC TCA GCG ATC TCG CCT | HSV-2 infected UW-268 cells |
|  | R: TAG CTG GAG AGT TTG ACC TTG TCG GTG |  |
| VZV | F: TCC GAC ATG CAG TCA ATT TCA ACG TC | VZV infected Batson cells |
|  | R: GGTCGG GTA GAC GCT ACC ACT CGT TT |  |
| EBV | F: CTT AGA ATG GTG GCCGGG CTG TAA AAT | EBV infected P3HR-1 cells |
|  | R: ATC CAG TAG GTC TTT GTG GAG CCC AAG |  |
| CMV | F: GCG CGT ACC GTT GAA AGA AAA GCA TAA | CMV infected AD169 cells |
|  | R: TGG GCA CTC GGG TCT TCA TCT CTT TAC |  |
| HHV-6 | F: ATG CGC CAT CAT AAT GCT CGG ATA CA | HHV-6 infected P3HR-1 cells |
|  | R: CCC TGC ATT CTT ACG GAA GCA AAA CG |  |

The primers and positive controls for the multiplex PCR　(Catalog No. 8665-6, SRL, Inc., Tokyo, Japan).

HSV-1: Herpes simplex virus-1, HSV-2: Herpes simplex virus, VZV: Varicella-zoster virus, EBV: Epstein-Barr virus, CMV: Cytomegalovirus, HHV-6: Human herpesvirus 6
